# Superficial capillary perfusion on optical coherence tomography angiography differentiates moderate and severe nonproliferative diabetic retinopathy

**DOI:** 10.1371/journal.pone.0240064

**Published:** 2020-10-22

**Authors:** Janice X. Ong, Changyow C. Kwan, Maria V. Cicinelli, Amani A. Fawzi

**Affiliations:** Department of Ophthalmology, Feinberg School of Medicine, Northwestern University, Chicago, IL, United States of America; Massachusetts Eye & Ear Infirmary, Harvard Medical School, UNITED STATES

## Abstract

**Purpose:**

To identify objective optical coherence tomography angiography (OCTA) parameters that characterize the spectrum of non-proliferative diabetic retinopathy (NPDR), especially those that distinguish moderate from severe NPDR.

**Methods:**

Sixty eyes of 60 patients with treatment-naïve NPDR (mild: 21, moderate: 21, severe: 18), 23 eyes with diabetes and no retinopathy, and 24 healthy control eyes were enrolled. OCTA slabs were segmented into superficial (SCP), middle (MCP), and deep capillary plexus (DCP) and thresholded by a new method based on DCP skeletonized vessel length. The foveal avascular zone (FAZ) area, parafoveal vessel density (VD), and adjusted flow index (AFI) from all three capillary layers and the vessel length density (VLD) of the SCP were compared between each severity group, after adjusting for age and image quality.

**Results:**

All vessel density markers decreased with increasing severity of NPDR. SCP VD and VLD demonstrated significant differences between eyes with diabetes with no retinopathy and mild NPDR (*p* = 0.001 and *p* < 0.001, respectively), as well as between moderate vs. severe NPDR (*p* = 0.004 and *p* = 0.009, respectively). MCP VD significantly decreased between moderate and severe NPDR (*p* = 0.01). AFI significantly increased in the SCP and showed a decreasing trend in the MCP and DCP with increasing NPDR severity.

**Conclusions:**

Changes in the SCP VD, SCP VLD, and MCP VD can distinguish severe NPDR from lower-risk stages. SCP changes may be more reliable due to their lower susceptibility to noise and projection artifacts. Thresholding OCTA images based on DCP skeletonized vessel length showed less variability in moderate and severe NPDR. Additional studies are warranted to validate this new thresholding method.

## Introduction

Diabetic retinopathy (DR) results from long-term effects of hyperglycemia on the microvasculature of the eye. DR affects one-third of the over 460 million diabetic patients worldwide and is one of the leading causes of vision loss in patients aged 20–74 [1, 2]. The pathology of early DR involves loss of pericytes and endothelial cells, resulting in capillary acellularity and diminished blood flow [3]. Eventually, retinal ischemia drives preretinal neovascularization through expression of angiogenic signaling molecules like vascular endothelial growth factor (VEGF), marking the transition from non-proliferative DR (NPDR) to proliferative DR (PDR) [4]. Potential complications of PDR include vitreous hemorrhage, traction retinal detachment, and neovascular glaucoma, leading to irreversible vision loss [5].

NPDR is further subclassified as mild, moderate, or severe based on clinical features like microaneurysms, hemorrhages, exudates and vascular abnormalities [6]. NPDR severity status is associated with the risk of further progression to PDR. While the one-year risk of progression to PDR is relatively low for patients with mild or moderate NPDR (5 and 15%, respectively), it is much higher in patients with severe and very severe NPDR, at 52% and 72%, respectively [7]. Despite the importance of NPDR staging for risk stratification and surveillance planning, current grading criteria are subjective, based on qualitative features, and are susceptible to clinician judgment and individual clinical presentation of the patient [8].

Optical coherence tomography angiography (OCTA) is a rapid, noninvasive imaging method that has been used to assess retinal perfusion in DR. The vascular changes on OCTA have been demonstrated to distinguish healthy eyes from eyes with diabetes and no DR, and to correlate with the severity of DR [9–11]. OCTA has also allowed characterization of the three distinct capillary plexuses in the macula (i.e., the superficial [SCP], the middle [MCP], and the deep capillary plexus [DCP]), which show unique pathologic changes in DR eyes [12–14]. To our knowledge, none of the prior studies have explored the three capillary plexuses across the different severity stages of NPDR. Furthermore, these studies have not distinguished treated and treatment-naïve eyes while studying NPDR severity. Moreover, the most sensitive thresholding algorithm to assess macular perfusion in DR remains a topic of debate [15, 16].

The purpose of this study is to characterize objective OCTA parameters across the entire spectrum of severity in treatment-naïve NPDR eyes. Additionally, we demonstrate the utility of a new thresholding method, which shows lower variability especially in the more advanced NPDR stages.

## Materials and methods

This prospective cross-sectional study enrolled healthy and diabetic patients seen between July 2017 and March 2020 at the Department of Ophthalmology at Northwestern University in Chicago, Illinois. The study was approved by the Institutional Review Board of Northwestern University and conducted in accordance with the tenets of the Declaration of Helsinki and the regulations of the Health Insurance Portability and Accountability Act. Written informed consent was obtained from all participants.

Study groups recruited were healthy patients with no retinal pathology (Healthy), diabetics with no apparent retinopathy (DM no DR), mild, moderate, and severe NPDR. Inclusion criteria were age between 25 and 75 years old, diagnosis of diabetes mellitus if applicable, and diagnosis of NPDR by a board-certified retina specialist. Color fundus photographs were obtained using the Ultra-widefield Scanning Laser Ophthalmoscope (Optomap Panoramic 200; Optos PLC, Scotland, U.K.) within 3 months of OCTA imaging.

Exclusion criteria were refractive error greater than 6.0 diopters, astigmatism greater than 3.0 diopters, significant medial or lens opacities, history of previous retinal surgery, central macular edema, and presence of other retinal vascular disease or glaucoma. Central macular edema was defined as central macular thickness greater than 320 μm for men and 305 μm for women on Spectralis OCT (Heidelberg Engineering Inc., Heidelberg, Germany) [17]. Only treatment-naïve eyes with no prior history of focal laser, pan-retinal photocoagulation (PRP), or intravitreal injections were considered, and only one eye per patient was included in the analysis.

Patients with NPDR were classified according to the International Clinical Diabetic Retinopathy Disease Severity Scale criteria as mild, moderate, or severe based on color fundus photographs evaluated by two independent graders (J.X.O. and C.C.K.) [8]. Disagreements between graders were decided by a third, senior grader (A.A.F.). In this staging system, eyes are considered to have severe NPDR if they have four quadrants of hemorrhages or microaneurysm, two quadrants of venous beading, or one quadrant of intra-retinal microvascular abnormalities (IRMA) exceeding standard reference photographs. Patients with hemorrhages/microaneurysms, soft exudates or venous beading not meeting severe NPDR criteria are staged as moderate NPDR, and patients with only microaneurysms are staged as mild NPDR.

Demographic and clinical data were obtained through electronic medical records. Age, gender, race, duration of diabetes, most recent hemoglobin A1c (HbA1c) within 6 months, and history of systemic hypertension were recorded for each patient.

### Sample size

Sample sizes were calculated using the G*Power free software package developed by Faul et al. [18]. We based our sample size calculation on capillary perfusion densities for SCP and DCP of the mild, moderate, and severe NPDR groups previously reported by Agemy et al. [19]. A Cohen’s *d* effect size was calculated for each group and used to determine the minimum sample size for comparisons between groups, assuming an α of 0.05 and a power of 0.8. Calculated minimum sample sizes for capillary perfusion density in the SCP were 6 for the mild vs. severe NPDR comparison, 6 for mild vs. moderate, and 5 for moderate vs. severe NPDR. Minimum sample sizes based on capillary perfusion density in the DCP were 7 for mild vs. severe, 25 for mild vs. moderate, and 58 for moderate vs. severe NPDR. Based on these calculations and the feasibility of recruiting patients in our practice, we chose a sample size of 20 subjects in each NPDR severity group.

### Image acquisition

OCTA images were obtained using the RTVue-XR Avanti system (Optovue Inc., Fremont, California, USA) with split-spectrum amplitude-decorrelation angiography (SSADA) algorithm [20]. Two consecutive B-scans, each containing 304 A-scans, were captured over a 3x3 mm^2^ region centered on the fovea. The A-scan rate was 70,000 scans/s, using a light source centered on 840 nm and a bandwidth of 45 nm. Information about angiographic flow was extracted from both B-scans with the SSADA algorithm. 3D Projection artifact removal (3D-PAR) technology by Optovue was applied to the images before extraction for further analysis. Only images with a quality index (Q-score) of 6 or greater and a signal strength index (SSI) of 50 or greater were included.

### Image analysis

#### Segmentation

Full retina and SCP angiograms were segmented automatically using the built-in AngioVue Analytics software (version 2017.1.0.151). The entire retinal slab was segmented from the internal limiting membrane (ILM) to 10 μm below the outer plexiform layer (OPL). The SCP was segmented from the ILM to 10 μm above the inner plexiform layer (IPL). Both the MCP and DCP were segmented manually as previously described, with the MCP as 10 μm above to 30 μm below the IPL, and the DCP as 30 μm below the IPL to 10 μm below the OPL [21].

#### Calculation of OCTA parameters

Quantitative parameters were determined for the parafoveal region, defined as the annulus with outer ring diameter 3 mm and inner ring diameter 1 mm around the center of the fovea. All image analysis was performed using ImageJ (developed by Wayne Rasband, National Institutes of Health, Bethesda, MD; available at http://rsb.info.nih.gov/ij/index.html). The vessel density (VD), the adjusted flow index (AFI), and the skeletonized vessel density, also known as vessel length density (VLD), were manually determined for each plexus. The area of the foveal avascular zone (FAZ) was also calculated from the full retinal slab by manual tracing as previously described [22].

Each slab was binarized and the percentage of the parafoveal area occupied by blood vessels was recorded as the VD. The AFI, which represents blood flow, was calculated as the average decorrelation value of all pixels above the noise threshold as previously described [21]. Binarized images were then skeletonized to determine the VLD using the Skeletonize (2D/3D) plugin for ImageJ (developed by Ignacio Arganda-Carreras; available at http://imagej.net/Skeletonize3D) [23]. This function reduces all vessel widths to 1 pixel and enables to eliminate the effects of the large vessels on vessel density in the SCP. The VLD was calculated as the ratio of vessel length in mm over the total area in mm^2^.

#### Binarization and thresholding methods

Initially, parafoveal vessel density was obtained for each image using the built-in AngioVue Analytics software [11, 21, 22]. However, using this approach in a pilot sample of randomly selected 8–9 eyes per study group (43 eyes in total) showed an increase in the DCP VD with increasing NPDR severity, a result that is contradictory to the pathophysiology of DR. Moreover, initial VD results also showed high variability within groups, especially in the DCP (S1 Fig).

We evaluated other potential thresholding methods by comparing the VD values obtained from the AngioVue software with two automated methods available in ImageJ, Mean and Huang, as well as a new method that we developed based on DCP skeletonized vessel length (described below) in the same subset of 43 eyes.

**Fig 1:**
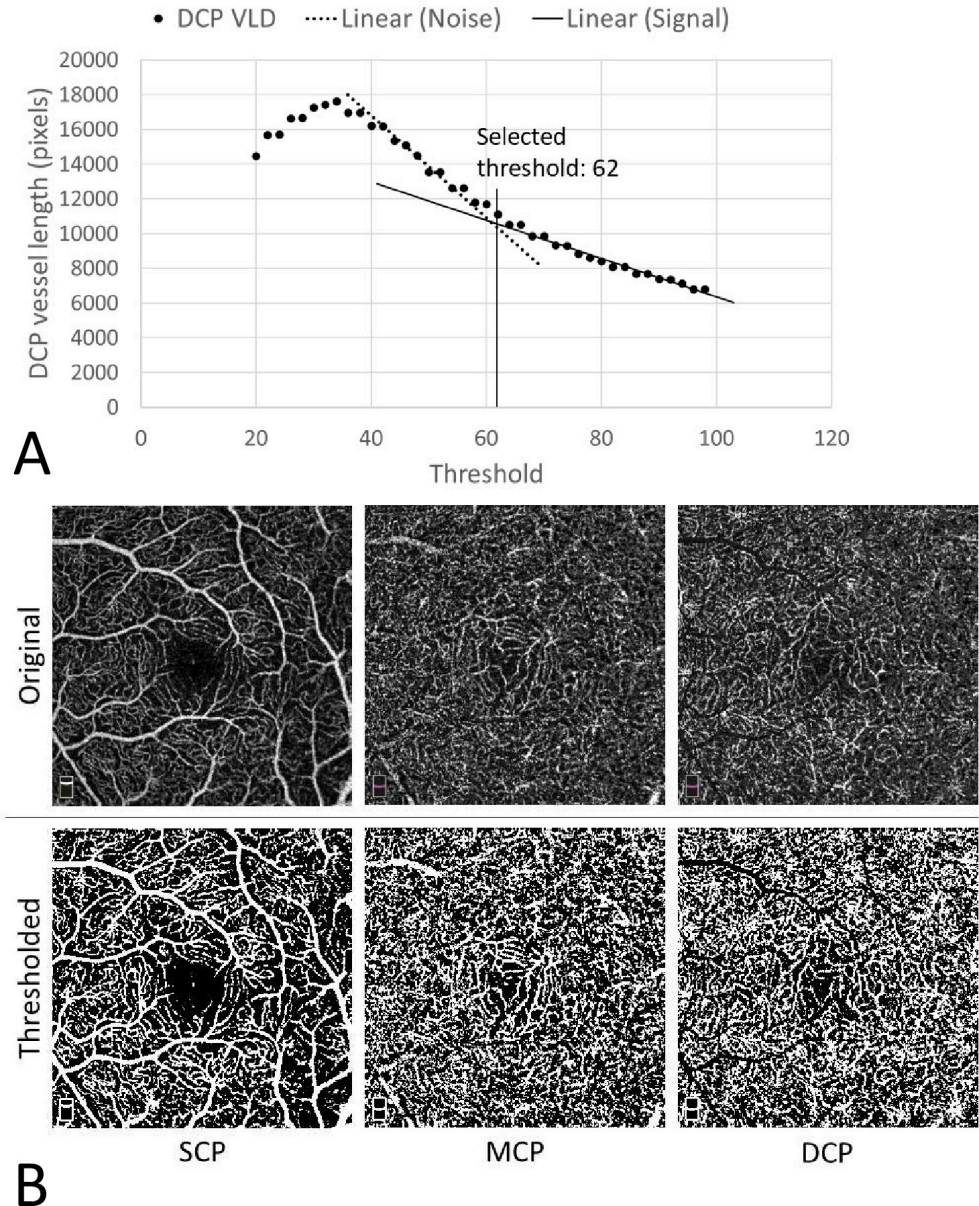
Demonstration of proposed deep capillary plexus (DCP) vessel length density (VLD)-based thresholding method. (A) OCTA images in the DCP were thresholded using progressively increasing empiric values and skeletonized vessel length calculated. To identify the optimum threshold value, we fitted regression lines to the steep and plateaued regions of the curve, which we hypothesized would represent noise and signal, respectively, and calculated the threshold value as the intersection of both regression lines. (B) Comparison of original images (top row) with thresholded images (bottom row).

Our proposed VLD-based thresholding method sought to define an objective cutoff that distinguishes true vessel signal from noise. Of the full retina and three capillary plexuses, the DCP parameters tended to show the most variability over the range of threshold values considered (S2 Fig). Therefore, we based our thresholding method on the plot of DCP signal vs. threshold (Fig 1). We hypothesized that as the chosen threshold value increased, the contribution of noise to the image would decrease at a faster rate than that of true vessel signal, so the threshold would mark the transition from steep to shallow curve. Therefore, we objectively placed the threshold at the point of transition of the signal vs. threshold curve in the DCP from steep to shallow.

To achieve this, we implemented a custom ImageJ macro to calculate total DCP VLD over a range of empiric binarization thresholds. We skeletonized the vessels to reduce the effects of noise at the vessel edges. For each binarization value, the macro extracted the DCP VLD as the number of pixels in the binarized, skeletonized DCP map. We then plotted the DCP VLDs against their corresponding empiric binarization values and drew best-fit lines on the resulting DCP VLD vs. threshold plot. We defined the signal-noise threshold as the transition from steep to plateau on this plot. To reduce the potential for subjectivity in interpretation of signal vs. noise, only the linear regions of the steep and plateau portions of the curve were considered when calculating the regression lines representing noise and signal, respectively. The final threshold value was chosen as the binarization value where the signal and noise best-fit lines intersected (Fig 1). All layers (SCP, MCP, and DCP) for each eye were binarized to the same threshold value for consistency. All subsequent thresholding and image analysis were done using this VLD-based method.

### Statistical analysis

Statistical analyses were performed using IBM SPSS statistics version 26 (IBM SPSS Statistics; IBM Corporation, Armonk, MY, USA). Two-tailed P-values of <0.05 were considered significant for all tests. Shapiro-Wilk tests were performed to determine whether demographic data were normally distributed. One-way ANOVA tests were then used to compare continuous demographic variables between the study groups. Categorical variables such as race, gender, type of DM, and diagnosis of hypertension were compared with chi-squared tests. Pearson and Spearman correlations were used to evaluate associations between OCTA parameters and potential confounding variables including age and Q-score.

Initially, Spearman rank correlations were used to evaluate monotonic associations between raw OCTA parameters and ordered disease stage (0 = Healthy, 1 = DM no DR, 2 = Mild NPDR, 3 = Moderate NPDR, 4 = Severe NPDR). To further resolve differences in OCTA parameters between individual disease groups, ANCOVA tests adjusted for the confounding effects of age and Q-score followed by post-hoc pairwise comparisons for OCTA parameters with a significant ANCOVA result were performed. P-values for post-hoc tests were adjusted using the Benjamini-Hochberg correction with false discovery rate of 0.05 to minimize both Type I and Type II errors [24]. We calculated effect sizes with Cohen’s *d* coefficient. Receiver operating characteristic (ROC) curves were generated for selected OCTA parameters to assess their diagnostic ability to identify severe NPDR. For ROC analyses, the area under curve (AUC), sensitivity, and specificity were determined from the ROC curve.

## Results

A total of 117 eyes of 117 patients meeting the initial inclusion and exclusion criteria were imaged. From the original sample of 117 eyes, 10 eyes were excluded for low OCTA image quality (Q-score < 6 or presence of significant motion or shadow artifacts in OCTA). Final groups were 24 eyes of healthy controls, 23 diabetic eyes without retinopathy, 21 eyes with mild NPDR, 21 eyes with moderate NPDR, and 18 eyes with severe NPDR. Study groups showed significant differences with respect to duration of diabetes, HbA1c, hypertension, and race (Table 1). All other demographic and clinical parameters were comparable between groups.

**Table 1:** Demographic and image characteristics of study patients in each diabetic retinopathy severity group.

Subject Characteristics	Healthy controls (n = 24)	DM no DR (n = 23)	Mild NPDR (n = 21)	Moderate NPDR (n = 21)	Severe NPDR (n = 18)
Male/female	9/15	8/15	8/13	8/13	6/12
Age (y), mean ± SD	46.54 ± 13.35	53.04 ± 13.99	52.71 ± 13.76	56.81 ± 10.88	55.94 ± 11.39
Race^‡^					
Caucasian	14 (58%)	14 (61%)	11 (52%)	6 (29%)	
African-American	4 (17%	4 (17%)	4 (19%)	9 (43%)	2 (11%)
Hispanic	6 (25%)	2 (9%)	3 (14%)	6 (29%)	16 (89%)
Asian		2 (9%)	2 (10%)		
Other/missing		1 (4%)	1 (5%)		
Refractive error (D), mean ± SD	-1.30 ± 1.83	-1.38 ± 2.08	-1.56 ± 1.82	-1.72 ± 2.46	-1.23 ± 1.49
DM type, I/II	–	6/17	8/13	7/14	1/17
Duration of DM* (y), mean ± SD	–	9.68 ± 6.80	15.91 ± 11.50	20.71 ± 10.85	20.22 ± 8.57
HbA1c* (%), mean ± SD	–	6.56 ± 0.90	7.25 ± 0.76	8.04 ± 1.66	8.42 ± 1.17
Hypertension^‡^	4 (17%)	11 (48%)	13 (62%)	10 (48%)	13 (72%)
Q-score*, mean ± SD	8.46 ± 0.83	8.17 ± 0.65	7.86 ± 0.85	7.57 ± 1.08	7.67 ± 0.77
Signal strength index*, mean ± SD	71.0 ± 6.3	68.1 ± 5.7	66.4 ± 6.8	64.1 ± 8.0	64.5 ± 6.3
Threshold, mean ± SD	54.5 ± 3.5	55.9 ± 3.1	56.3 ± 4.9	55.7 ± 4.7	57.8 ± 4.4

* Statistically significant at 0.05 level (two-tailed) with ANOVA analysis.

^‡^ Statistically significant at 0.05 level (two-tailed) with Χ^2^ analysis.

Abbreviations: DM = diabetes mellitus; DR = diabetic retinopathy; NPDR = non-proliferative diabetic retinopathy.

### Evaluation of thresholding methods

We first sought to address the potential concern that VLD-based thresholding could introduce bias related to severity stage, potentially confounding the calculated OCTA parameters. One-way ANOVA of mean threshold showed no difference between severity groups (*p* = 0.163), indicating that our threshold calculation method was not biased by disease status (Table 1).

To compare our method to the others, we calculated average VD with each method (AngioVue, automated Mean, automated Huang, and VLD-based) in the SCP, MCP, and DCP for the same pilot sample of 8–9 eyes per study group, 43 eyes total. The AngioVue built-in values, automated Huang, and automated Mean methods all demonstrated an apparent rise in DCP VD with increasing disease severity, a finding that contradicts the known pathology of capillary dropout in DR and the evident nonperfusion seen in the raw images (S1 Fig). Large standard deviations were also seen for these three methods, especially in the moderate and severe NPDR patients. We had no access to the binarized images from the AngioVue software to evaluate the accuracy of binarization in their VD calculations. Our qualitative evaluation of the automated Mean and Huang methods showed their tendency to over-estimate VD in the DCP in eyes with severe NPDR compared to the original OCTA images (S3 Fig). In contrast, the DCP VLD-based thresholding method was less likely to generate vessels in non-perfused areas and resolved the apparent anomalous trend in DCP compared to built-in and automated Mean or Huang thresholding. Based on these data, we performed all subsequent image analysis with the DCP-VLD-based thresholding.

### Comparison of OCTA parameters between disease groups

**Fig 2:**
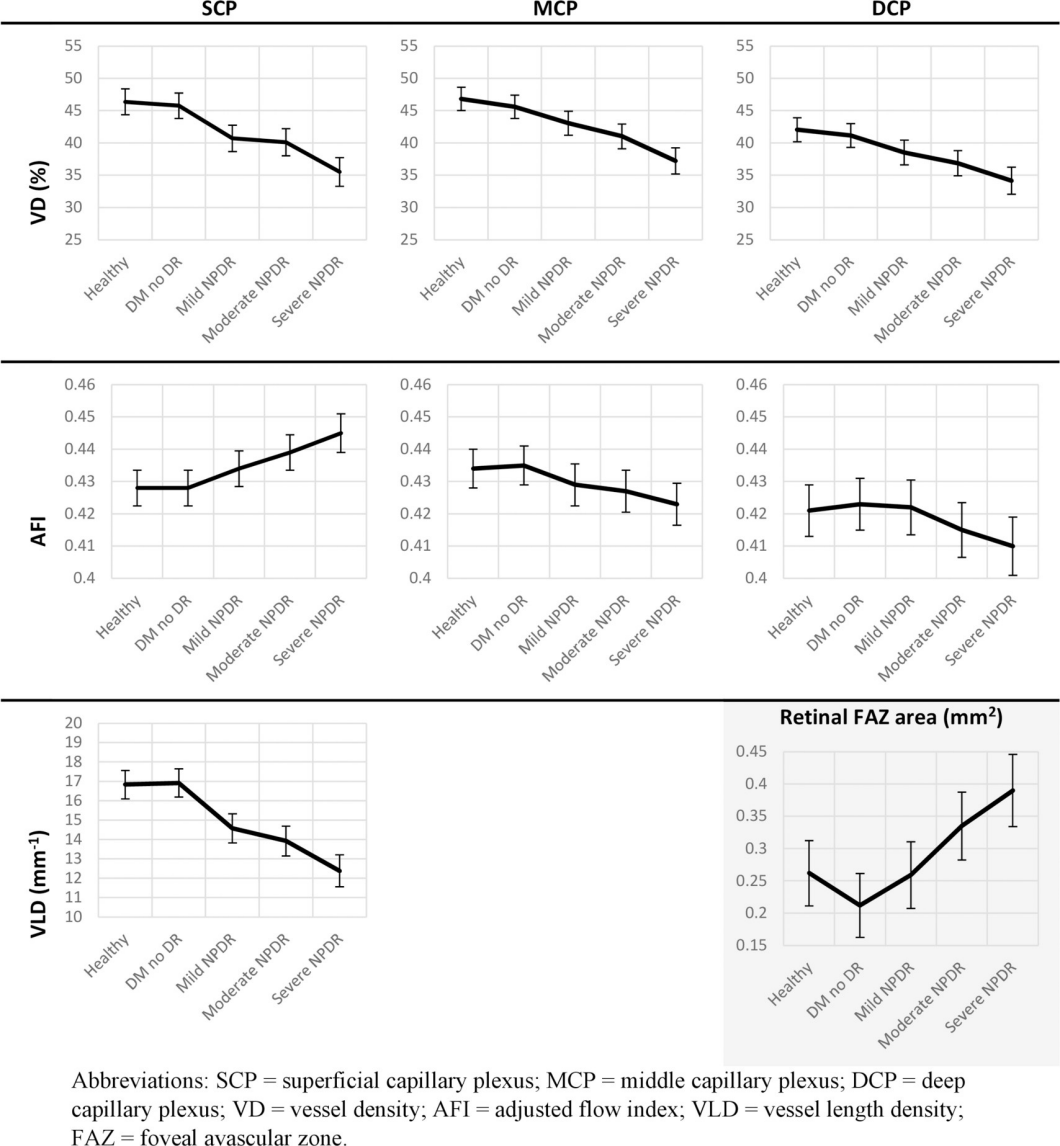
Optical coherence tomography angiography capillary parameters plotted by diabetic retinopathy severity group. Data were adjusted for age and image quality as assessed by Q-score. Columns from left to right: superficial (SCP), middle (MCP) and deep capillary plexus (DCP). Rows from top to bottom: mean values for parafoveal vessel density (VD), adjusted flow index (AFI), and vessel length density (VLD; only applicable for SCP), graphed continuously across healthy controls, diabetic eyes without retinopathy, mild NPDR, moderate NPDR, and severe NPDR. Foveal avascular zone area (FAZ) was calculated from the full retinal slab and is shown in the bottom right graph. Error bars represent 95% confidence intervals.

Overall, the SCP VLD and VD in all three capillary plexuses progressively declined with increasing NPDR severity stage, while the FAZ area increased (Table 2, Fig 2). The SCP AFI showed a moderate positive correlation with increasing NPDR severity (*R*s = 0.381, *p* < 0.001). Conversely, MCP AFI (Spearman rank correlation *R*s = -0.445, *p* < 0.001) and DCP AFI (*R*s = -0.329, *p* < 0.001) correlated negatively with the NPDR severity classes.

**Table 2:** Association of optical coherence tomography angiography parameter values across non-proliferative diabetic retinopathy severity groups using Spearman correlations.

Parameter	Spearman *R-*value	Spearman *p*-value
VD		
SCP	-0.654	*p* < 0.001
MCP	-0.660	*p* < 0.001
DCP	-0.610	*p* < 0.001
VLD		
SCP	-0.713	*p* < 0.001
AFI		
SCP	0.381	*p* < 0.001
MCP	-0.445	*p* < 0.001
DCP	-0.329	*p* < 0.001
FAZ		
Retina	0.374	*p* < 0.001

Abbreviations: SCP = superficial capillary plexus; MCP = middle capillary plexus; DCP = deep capillary plexus; VD = vessel density; AFI = adjusted flow index; VLD = vessel length density; FAZ = foveal avascular zone.

After adjusting for age and Q-score in ANCOVA analyses (Table 3), VD in all three plexuses, SCP VLD, FAZ, and SCP AFI remained significant, while MCP AFI and DCP AFI were marginally (*p* = 0.078) and not significant (*p* = 0.207), respectively.

**Table 3:** ANCOVA analysis of age- and Q-score-adjusted optical coherence tomography angiography capillary parameters by diabetic retinopathy severity group.

Parameters, Mean ± SD	Healthy controls (n = 24)	DM no DR (n = 23)	Mild NPDR (n = 21)	Moderate NPDR (n = 21)	Severe NPDR (n = 18)	ANCOVA
VD (%)						
SCP	46.38 ± 4.93	45.76 ± 4.77	40.72 ± 4.74	40.12 ± 4.84	35.52 ± 4.79	*p* < 0.001
MCP	46.83 ± 4.48	45.61 ± 4.34	43.05 ± 4.31	41.03 ± 4.40	37.23 ± 4.35	*p* < 0.001
DCP	42.05 ± 4.60	41.15 ± 4.45	38.52 ± 4.42	36.87 ± 4.51	34.16 ± 4.46	*p* < 0.001
VLD (mm^-1^)						
SCP	16.84 ± 1.82	16.92 ± 1.76	14.58 ± 1.75	13.92 ± 1.78	12.39 ± 1.76	*p* < 0.001
AFI						
SCP	0.428 ± 0.015	0.428 ± 0.014	0.434 ± 0.014	0.439 ± 0.014	0.445 ± 0.013	*p* < 0.001
MCP	0.434 ± 0.015	0.435 ± 0.014	0.429 ± 0.014	0.427 ± 0.014	0.423 ± 0.013	*p* = 0.078
DCP	0.421 ± 0.020	0.423 ± 0.019	0.422 ± 0.018	0.415 ± 0.018	0.410 ± 0.021	*p* = 0.207
FAZ (mm^2^)						
Retina	0.262 ± 0.122	0.212 ± 0.120	0.259 ± 0.119	0.335 ± 0.124	0.390 ± 0.119	*p* < 0.001

Abbreviations: SCP = superficial capillary plexus; MCP = middle capillary plexus; DCP = deep capillary plexus; VD = vessel density; AFI = adjusted flow index; VLD = vessel length density; FAZ = foveal avascular zone.

Results of pairwise comparisons between groups are summarized in Table 4. Between-group comparisons differing by only one stage of disease severity showed that SCP VD had a progressive stepwise decrease, with the biggest differences occurring in transition from DM no DR to mild NPDR (*p* = 0.001) and from moderate NPDR to severe NPDR (*p* = 0.004). SCP VLD followed a similar stepwise pattern, with the greatest differences between DM no DR and mild NPDR (*p* < 0.001) and between moderate and severe NPDR (*p* = 0.009). MCP VD and DCP VD both exhibited consistent decrease with disease severity. Change in MCP VD was significant at the moderate to severe NPDR transition (*p* = 0.010) and marginally significant at the DM no DR to mild NPDR transition (*p* = 0.068). DCP VD showed no significant differences between adjacent disease stages.

**Table 4:** Pairwise comparisons of optical coherence tomography angiography capillary parameters between diabetic retinopathy severity groups using ANCOVA post-hoc analysis.

Parameter	Comparison	Healthy controls (n = 24)	DM no DR (n = 23)	Mild NPDR (n = 21)	Moderate NPDR (n = 21)
SCP VD					
	vs. DM no DR	*p* = 0.731			
	vs. Mild NPDR	*p* < 0.001*	*p* = 0.001*		
	vs. Moderate NPDR	*p* < 0.001*	*p* < 0.001*	*p* = 0.685	
	vs. Severe NPDR	*p* < 0.001*	*p* < 0.001*	*p* = 0.001*	*p* = 0.004*
MCP VD					
	vs. DM no DR	*p* = 0.338			
	vs. Mild NPDR	*p* = 0.009*	*p* = 0.068		
	vs. Moderate NPDR	*p* < 0.001*	*p* = 0.002*	*p* = 0.148	
	vs. Severe NPDR	*p* < 0.001*	*p* < 0.001*	*p* < 0.001*	*p* = 0.010*
DCP VD					
	vs. DM no DR	*p* = 0.491			
	vs. Mild NPDR	*p* = 0.018*	*p* = 0.076		
	vs. Moderate NPDR	*p* = 0.001*	*p* = 0.006*	*p* = 0.256	
	vs. Severe NPDR	*p* < 0.001*	*p* < 0.001*	*p* = 0.006*	*p* = 0.074
SCP VLD					
	vs. DM no DR	*p* = 0.872			
	vs. Mild NPDR	*p* < 0.001*	*p* < 0.001*		
	vs. Moderate NPDR	*p* < 0.001*	*p* < 0.001*	*p* = 0.255	
	vs. Severe NPDR	*p* < 0.001*	*p* < 0.001*	*p* < 0.001*	*p* = 0.009*
SCP AFI					
	vs. DM no DR	*p* = 0.980			
	vs. Mild NPDR	*p* = 0.209	*p* = 0.224		
	vs. Moderate NPDR	*p* = 0.025*	*p* = 0.025*	*p* = 0.250	
	vs. Severe NPDR	*p* = 0.001*	*p* = 0.001*	*p* = 0.025*	*p* = 0.204
FAZ					
	vs. DM no DR	*p* = 0.206			
	vs. Mild NPDR	*p* = 0.949	*p* = 0.221		
	vs. Moderate NPDR	*p* = 0.090	*p* = 0.004*	*p* = 0.084	
	vs. Severe NPDR	*p* = 0.003*	*p* < 0.001*	*p* = 0.005*	*p* = 0.218

*P*-values were adjusted for multiple comparisons using the Benjamini-Hochberg correction with false discovery rate set at 0.05.

* Statistical significance (adjusted *p*-value < 0.05).

Abbreviations: SCP = superficial capillary plexus; MCP = middle capillary plexus; DCP = deep capillary plexus; VD = vessel density; AFI = adjusted flow index; VLD = vessel length density; FAZ = foveal avascular zone.

**Fig 3:**
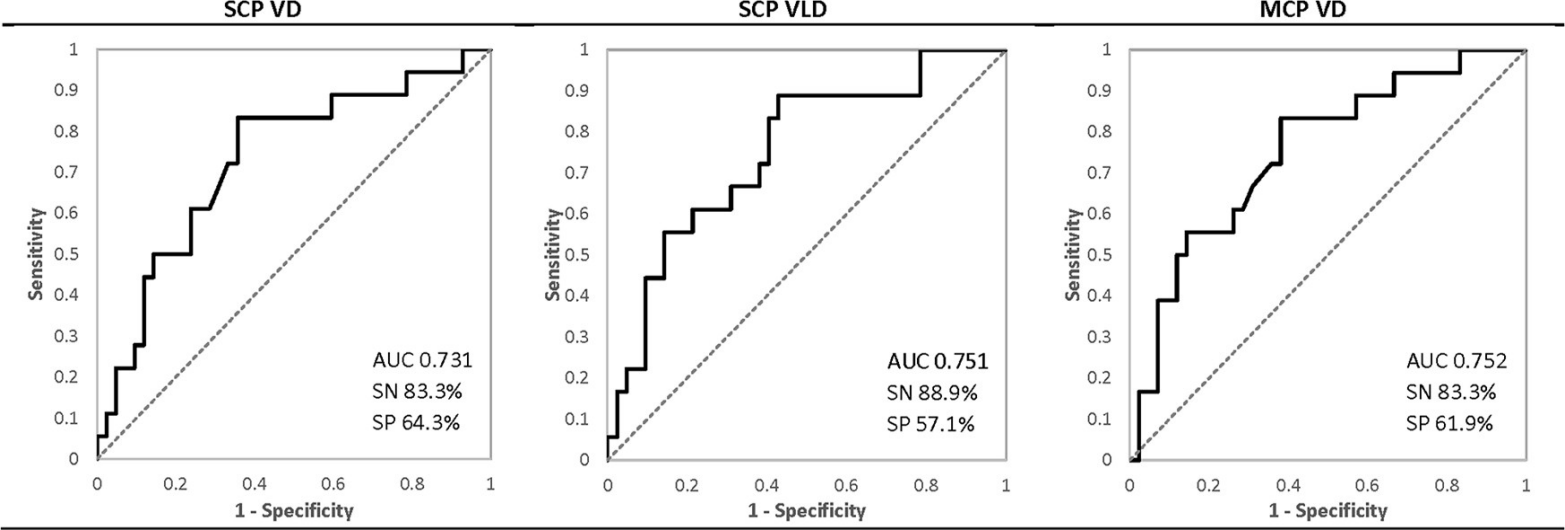
Receiver operating characteristic curves for optical coherence tomography angiography parameters distinguishing severe from mild and moderate NPDR. ROC analysis was performed to assess the ability of OCTA parameters to distinguish severe from mild and moderate NPDR. Superficial capillary plexus (SCP) vessel density (VD), vessel length density (VLD), and middle capillary plexus (MCP) VD were analyzed. AUC = area under curve; SN = sensitivity; SP = specificity.

Based on pairwise comparison data for moderate vs. severe NPDR, ROC analysis was performed for SCP VD, SCP VLD, and MCP VD to assess the diagnostic ability of these parameters in distinguishing severe NPDR from mild and moderate NPDR. Fig 3 summarizes the ROC analysis data. The three parameters showed AUC values ranging from 0.731 to 0.752, with corresponding sensitivities from 83.3% to 88.9% and specificities from 57.1% to 64.3%.

All NPDR groups (mild, moderate, and severe) differed significantly from healthy controls in terms of VD in all three capillary plexuses. Compared to DM no DR, moderate and severe NPDR patients showed significantly lower VD values in all three capillary plexuses; mild NPDR patients were only distinguished from DM no DR by SCP VD and SCP VLD.

There were no statistically significant differences between adjacent stages for SCP AFI or FAZ area, although an overall increase with disease severity was seen for both parameters.

## Discussion

In this cross-sectional study, we used OCTA imaging to characterize microvascular changes in treatment-naïve eyes of diabetic subjects across NPDR severity stages. We especially sought to identify parameters that could serve as biomarkers for the distinction of severe NPDR from other stages, as patients with severe NPDR are at higher risk for progression to PDR. We identified three OCTA parameters in two different capillary plexuses—SCP VD, SCP VLD, and MCP VD—that quantitatively differentiated moderate from severe NPDR. Our study is uniquely positioned to evaluate the transition from moderate to severe NPDR. Previous studies evaluating DR severity on OCTA have either pooled patients with NPDR or evaluated only a single stage of retinopathy [11, 12, 14, 25, 26]. Others have combined moderate NPDR with either the mild or severe NPDR groups for analysis, which limits the ability to resolve differences between stages and precludes comparing severe NPDR against other stages [9, 10, 27]. In contrast to our study, prior studies that have analyzed all three NPDR stages have either been limited by low patient number (partially mitigated by imaging both eyes of the same patient) or including previously treated eyes [19, 28–31].

Overall, we found that SCP VD and VLD decreased with increasing NPDR severity, with statistically significant differences in the DM no DR to mild NPDR and moderate to severe NPDR transitions (Fig 2, Table 4). MCP VD also differed significantly between moderate and severe NPDR. DCP VD was only significant between groups separated by two or more levels of disease severity, suggesting that changes in the SCP can be more sensitive for staging NPDR.

There are several potential considerations to explain why the SCP could better distinguish NPDR stages in our study. The SCP is less affected by the noise and projection artifacts that plague OCTA measurements in the deeper retinal layers. Since the deeper layers are more susceptible to projection artifacts and signal attenuation, this could potentially explain greater variation in calculated OCTA parameters in the MCP and DCP [32]. Similar to our study, Durbin et al. found SCP VD and perfusion density to have greater diagnostic value than the DCP across patients with pre-DR, mild NPDR, and combined moderate/severe NPDR [9]. They found that the SCP continued to have greater diagnostic value even after DCP image quality was improved by removal of decorrelation tail projection artifacts. Although our current study images underwent projection artifact removal, theoretically minimizing decorrelation tails, the effects of artifacts and signal attenuation may have limited our ability to completely resolve the DCP [33].

Our study was also specifically powered to detect differences in the SCP, further limiting our ability to assess potential differences in the DCP. Comparing the DCP VD data for DM no DR vs. mild NPDR and moderate vs. severe NPDR yielded moderate effect sizes (Cohen’s *d* = 0.593 and 0.604, respectively), confirming our original sample size calculations and the need for a larger sample size (44–46 patients per group) to detect true differences in the DCP. Overall, our data suggest that the SCP and MCP may be most practical and effective in quantifying microvascular changes occurring in NPDR, especially when sample size is a limitation.

Although ours and several other studies have found the SCP to best correlate with disease severity, the relative extent of superficial vs. deep changes with increasing NPDR severity is still debated [9–11, 19, 26, 28, 30, 31]. For example, Sambhav et al. found changes in DCP perfusion to be earlier and more pronounced with increasing NPDR severity, while Li et al. found both SCP and DCP VD changes to be significant [30, 31]. One potential source of discrepancy between these studies and ours is their segmentation of OCTA images into only SCP and DCP without separate consideration of the MCP. Partial incorporation of the anatomic MCP into either the SCP or DCP may confound interpretation of OCTA data across different machines and segmentation regimes [12, 34]. We found that with progressive NPDR stages, changes in the MCP tended to follow those in the DCP (Fig 2), a result consistent with previous studies that analyzed the MCP separately [12–14].

While previous studies have used AngioVue built-in software values or automated thresholding methods to determine VD, the use of a novel image thresholding method may also account for differences in our results [22, 30, 31]. Among the ImageJ automated thresholding methods, the Mean method has been previously demonstrated to have high reproducibility for thresholding OCTA images of the SCP [15]. However, we found large standard deviations when using the AngioVue proprietary algorithm DCP VD values, as well as when using automated thresholding calculations in moderate and severe NPDR images (S1 Fig). This to us would suggest that these methods introduce high variability, especially in eyes with relatively low vessel density (S3 Fig). We believe our findings highlight one potential source for disparate conclusions regarding the DCP parameters in previous studies, especially in studies with small sample size and those using built-in software VD values or automated thresholding methods. As shown by our thresholding data (S1 and S3 Figs), we found that our newly described DCP VLD-based thresholding more faithfully reproduced the true DCP vessel density, especially in eyes with moderate to severe NPDR where vessel dropout is more pronounced. In contrast, we observed that the AngioVue built-in and other automated thresholding methods tend to overestimate vessel density in moderate and severe NPDR eyes. Regarding its feasibility of use, the DCP VLD-based method uses existing functions available in ImageJ, a publicly available, open-source software, and as we have demonstrated, can be partially automated using macros for more efficient image processing.

To further evaluate the diagnostic accuracy of our parameters for severe NPDR compared to mild and moderate NPDR, we performed ROC analysis for the SCP VD, VLD and MCP VD. The analysis yielded moderate AUC values (0.731–0.752) with sensitivity ranging from 83.3% to 88.9% and specificity ranging from 57.1% to 64.3% (Fig 3). Durbin et al. did not perform comparisons between adjacent NPDR severity stages, leaving the most clinically relevant transitions in disease progression an unanswered question [9]. Ashraf et al. used a statistical model combining multiple OCTA parameters to distinguish PDR from NPDR with sensitivity of 86% and specificity of 70% but did not perform any analysis within NPDR stages [22]. We achieved comparable sensitivity but lower specificity, suggesting that these SCP and MCP parameters may provide useful information for screening severe NPDR.

Our finding that SCP AFI, a surrogate for blood flow, significantly increases with NPDR severity (Fig 2) supports previously published data from our group, which demonstrated an increase in age- and sex-adjusted SCP AFI with increasing DR severity in patients with NPDR and PDR [12]. Our raw data showed a moderately negative correlation between MCP and DCP AFI and increasing DR severity (Table 2), but these differences became insignificant after adjusting for potential confounders. Combined with the VD data, the AFI data suggest that with increasing DR severity, there is increased blood flow through fewer vessels in the SCP in conjunction with decreased flow and fewer capillaries in the MCP and DCP. These opposite effects could result from autoregulatory dysfunction of the retinal capillaries, or a progressive “steal” phenomenon by the dilated telangiectatic SCP capillaries, as we have previously suggested [12, 35].

We found no significant differences in OCTA parameters between healthy eyes and diabetic eyes without retinopathy. In contrast, Rosen et al. reported increased capillary density in the full retina in eyes with no DR compared to healthy eyes [36]. In contrast to our methodology, these comparisons were significant only in the 200 μm annulus directly surrounding the FAZ, while our study evaluated the entire area of the *en face* 3×3 mm^2^ scan, which may have limited our ability to resolve local changes.

We also observed a significant increase in size of the FAZ with increasing NPDR severity (Fig 2, Table 2), which is consistent with results from prior studies [37, 38]. However, we found that FAZ area alone was insufficient to distinguish adjacent stages of NPDR (Table 4), likely due to the individual variation in FAZ size that exists even in healthy individuals [39, 40].

The strengths of our study include the focus on the entire spectrum of severity of NPDR, the exclusion of previously treated eyes, the analysis of all three capillary plexuses of the retina, and the use of a new thresholding method that makes a quantitative distinction between signal and noise. By excluding eyes with central diabetic macular edema, we avoided the potential confounding effects of relevant artifacts and segmentation errors on our results [41].

Limitations of our study include the inadequate powering of the study for the DCP, which may have limited our ability to resolve true differences in DCP parameters between groups, and the significant differences in racial composition between study groups. The moderate NPDR group had a higher proportion of African-American patients relative to the other groups, while the severe NPDR group was disproportionately Hispanic. Chun et al. demonstrated that OCTA parameters may vary significantly with race in healthy subjects, with African-American patients having lower baseline DCP parafoveal VD and higher FAZ area compared to white patients [42]. While we do not know of any influence of Hispanic race on vascular parameters, larger datasets are needed to explore race as a potential confounder. Additionally, while univariate analysis showed that there was a non-significant difference in diabetes type between groups overall (*p* = 0.073), we noted that the severe NPDR group had a relatively lower fraction of type I DM.

The cross-sectional design also limits our ability to predict progression over time in an individual patient, which would be an important future longitudinal study. Additionally, while the VLD-based thresholding method we proposed is based on a quantitative distinction between signal and noise, theoretically making it less susceptible to the effects of foveal artifacts or non-perfused but noisy areas, it may also artifactually remove low-perfusion vessels with pixel intensity close to background noise. Further validation of this method in patients with a broader range of diagnoses and image qualities will be needed to confirm its generalizability.

In conclusion, our study demonstrates that vessel density in the SCP and MCP exhibit the most significant changes as NPDR severity advances. We found that SCP VD, SCP VLD, and MCP VD showed significant differences between eyes with moderate and severe NPDR. Our results suggest that changes in the SCP visualized through OCTA may facilitate distinguishing patients with severe NPDR from those with lower-risk NPDR. Future studies are needed to validate our new thresholding algorithm as well as longitudinal studies to explore the potential predictive power of the SCP for DR progression.

## Supporting information

S1 Fig Pilot data comparing superficial (SCP), middle (MCP), and deep (DCP) vessel densities (VD) determined by four different thresholding methods in patients with varying NPDR severity. 8–9 eyes from each level of NPDR severity (43 eyes in total) were selected and the DCP VD was determined using AngioVue built-in software values, automated Huang, automated Mean, and DCP VLD-based thresholding. Error bars represent 1 SD.
(DOCX)

S2 Fig Variation in calculated values of vessel density and adjusted flow index over a range of threshold values for a single eye. The vessel density (VD), adjusted flow index (AFI), and skeletonized vessel density (VLD) were calculated for the full retinal slab (Retina), superficial (SCP), middle (MCP), and deep capillary plexuses (DCP) over a range of empiric threshold values. The calculated values of these parameters were plotted against the corresponding threshold values to evaluate changes with increasing threshold.
(DOCX)

S3 Fig Comparison of thresholding methods for deep capillary plexus (DCP) vessel density across increasing levels of NPDR severity in representative eyes. The DCP slabs from representative eyes for each stage of NPDR were selected and binarized according to automated Huang, automated Mean, or DCP VLD-based thresholding. Binarized images were compared to original images. Selected areas of nonperfusion are indicated by red arrows.
(DOCX)

S1 Dataset Raw demographic, clinical, and optical coherence tomography angiography data. (XLSX)
