# Supplementary material for: Superficial capillary perfusion on optical coherence tomography angiography differentiates moderate and severe nonproliferative diabetic retinopathy
Source: PLoS One. 2020 Oct 22;15(10):e0240064. doi: 10.1371/journal.pone.0240064 (PMC7580912; doi:10.1371/journal.pone.0240064)
Supplement: S1 Fig — 8–9 eyes from each level of NPDR severity (43 eyes in total) were selected and the DCP VD was determined using AngioVue built-in software values, automated Huang, automated Mean, and DCP VLD-based thresholding. Error bars represent 1 SD. (DOCX) [file pone.0240064.s001.docx]

|  | **SCP** | **MCP** | **DCP** |
| --- | --- | --- | --- |
| **AngioVue software** |  |  |  |
| **Automated (Huang)** |  |  |  |
| **Automated (Mean)** |  |  |  |
| **DCP VLD-based** |  |  |  |

**S1 Fig.** **Pilot data comparing superficial (SCP), middle (MCP), and deep (DCP) vessel densities (VD) determined by four different thresholding methods in patients with varying NPDR severity.** 8-9 eyes from each level of NPDR severity (43 eyes in total) were selected and the DCP VD was determined using AngioVue built-in software values, automated Mean, automated Huang, and DCP VLD-based thresholding. Error bars represent 1 SD.
