# Supplementary material for: Superficial capillary perfusion on optical coherence tomography angiography differentiates moderate and severe nonproliferative diabetic retinopathy
Source: PLoS One. 2020 Oct 22;15(10):e0240064. doi: 10.1371/journal.pone.0240064 (PMC7580912; doi:10.1371/journal.pone.0240064)
Supplement: S2 Fig — The vessel density (VD), adjusted flow index (AFI), and skeletonized vessel density (VLD) were calculated for the full retinal slab (Retina), superficial (SCP), middle (MCP), and deep capillary plexuses (DCP) over a range of empiric threshold values. The calculated values of these parameters were plotted against the corresponding threshold values to evaluate changes with increasing threshold. (DOCX) [file pone.0240064.s002.docx]

**S2 Fig. Variation in calculated values of vessel density and adjusted flow index over a range of threshold values for a single eye.** The vessel density (VD), adjusted flow index (AFI), and skeletonized vessel density (VLD) were calculated for the full retinal slab (Retina), superficial (SCP), middle (MCP), and deep capillary plexuses (DCP) over a range of empiric threshold values. The calculated values of these parameters were plotted against the corresponding threshold values to evaluate changes with increasing threshold.
