# Supplementary material for: Superficial capillary perfusion on optical coherence tomography angiography differentiates moderate and severe nonproliferative diabetic retinopathy
Source: PLoS One. 2020 Oct 22;15(10):e0240064. doi: 10.1371/journal.pone.0240064 (PMC7580912; doi:10.1371/journal.pone.0240064)
Supplement: S3 Fig — The DCP slabs from representative eyes for each stage of NPDR were selected and binarized according to automated Huang, automated Mean, or DCP VLD-based thresholding. Binarized images were compared to original images. Selected areas of nonperfusion are indicated by red arrows. (DOCX) [file pone.0240064.s003.docx]

**
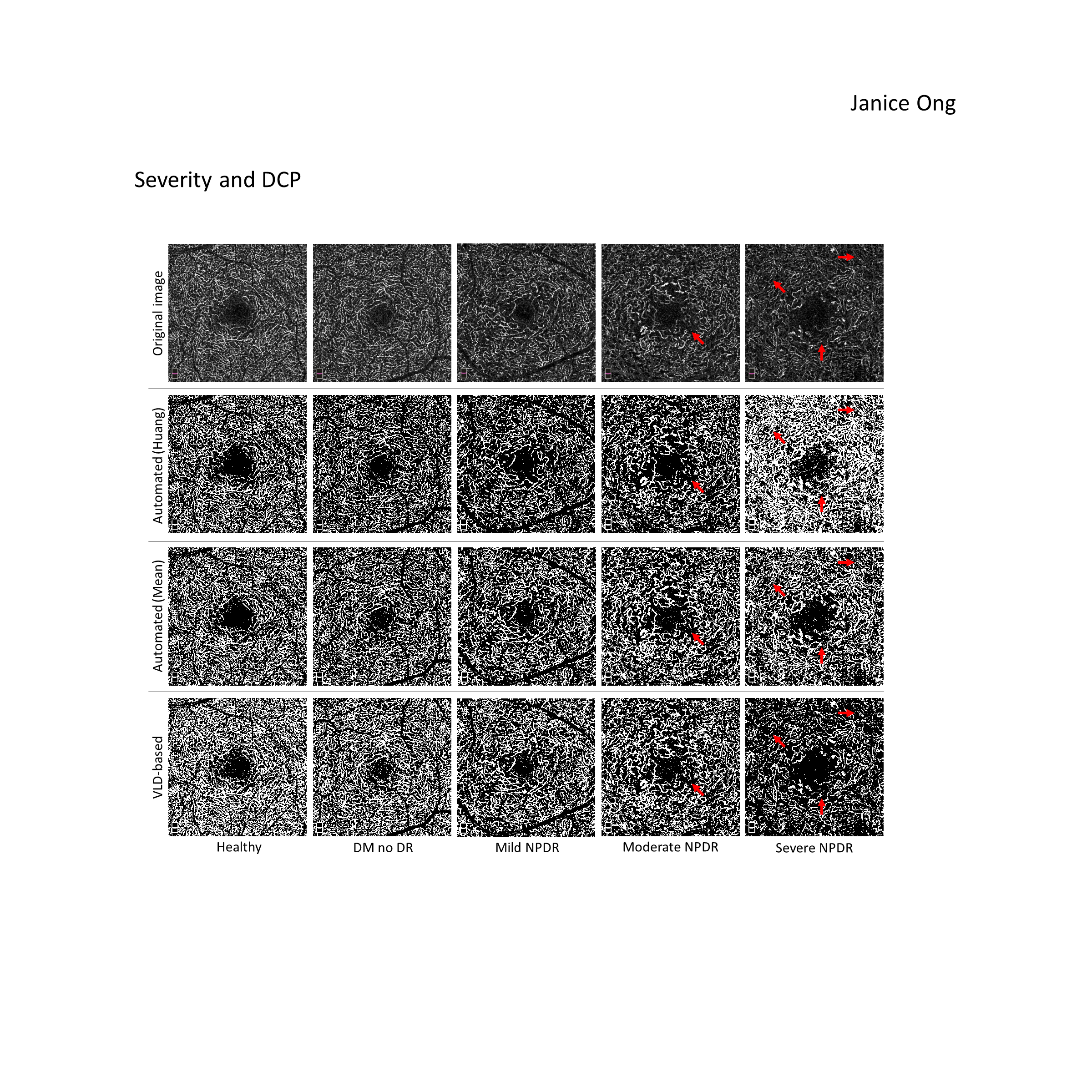
**

**S3 Fig. Comparison of thresholding methods for deep capillary plexus (DCP) vessel density across increasing levels of NPDR severity in representative eyes.** The DCP slabs from representative eyes for each stage of NPDR were selected and binarized according to automated Huang, automated Mean, or DCP VLD-based thresholding. Binarized images were compared to original images. Selected areas of nonperfusion are indicated by red arrows.
